# Amygdala activity contributes to the dissociative effect of cannabis on pain perception

**DOI:** 10.1016/j.pain.2012.09.017

**Published:** 2013-01

**Authors:** Michael C. Lee, Markus Ploner, Katja Wiech, Ulrike Bingel, Vishvarani Wanigasekera, Jonathan Brooks, David K. Menon, Irene Tracey

**Affiliations:** a Centre for Functional MRI of the Brain (FMRIB), Department of Clinical Neurology and Nuffield Department of Clinical Neurosciences, Division of Anaesthesia, University of Oxford, Oxford, UK; b Department of Neurology, Technische Universtität München, Munich, Germany; c NeuroImage Nord, Department of Neurology, University Medical Center, Hamburg-Eppendorf, Germany; d Department of Medicine, Division of Anaesthesia, University of Cambridge, Cambridge, UK

**Keywords:** Cannabinoids, Humans, Pain, Hyperalgesia, fMRI, Brain, Amygdala

## Abstract

Cannabis is reported to be remarkably effective for the relief of otherwise intractable pain. However, the bases for pain relief afforded by this psychotropic agent are debatable. Nonetheless, the frontal-limbic distribution of cannabinoid receptors in the brain suggests that cannabis may target preferentially the affective qualities of pain. This central mechanism of action may be relevant to cannabinoid analgesia in humans, but has yet to be demonstrated. Here, we employed functional magnetic resonance imaging to investigate the effects of delta-9-tetrahydrocannabinol (THC), a naturally occurring cannabinoid, on brain activity related to cutaneous ongoing pain and hyperalgesia that were temporarily induced by capsaicin in healthy volunteers. On average, THC reduced the reported unpleasantness, but not the intensity of ongoing pain and hyperalgesia: the specific analgesic effect on hyperalgesia was substantiated by diminished activity in the anterior mid cingulate cortex. In individuals, the drug-induced reduction in the unpleasantness of hyperalgesia was positively correlated with right amygdala activity. THC also reduced functional connectivity between the amygdala and primary sensorimotor areas during the ongoing-pain state. Critically, the reduction in sensory-limbic functional connectivity was positively correlated with the difference in drug effects on the unpleasantness and the intensity of ongoing pain. Peripheral mechanisms alone cannot account for the dissociative effects of THC on the pain that was observed. Instead, the data reveal that amygdala activity contributes to interindividual response to cannabinoid analgesia, and suggest that dissociative effects of THC in the brain are relevant to pain relief in humans.

## Summary

fMRI evidence suggests that delta-9-tetrahydrocannabinol, the psychoactive component of cannabis, targets the affective aspects of pain in humans by altering the activity and functional connections of the amygdala.

## 1 Introduction

Delta-9-tetrahydrocannabinol (THC), a partial cannabinoid-1 (CB-1) receptor agonist, can relieve chronic pain that is often refractory to conventional analgesics [4]. The neural bases of such pain relief remain unclear in humans, even though peripheral and central mechanisms for cannabinoid analgesia have been demonstrated in other species [39]. However, recent evidence reveals that CB-1 receptors densely populate frontal-limbic compared to somatosensory areas in the human cortex [8,13,21]. This distribution of CB-1 receptors in the brain suggests that THC may target the affective qualities of pain specifically, given that the affective-motivational and sensory aspects of pain are known to be separable functions of frontal-limbic and somatosensory cortices, respectively [2,6,47]. Of the limbic areas, data from preclinical studies clearly demonstrate that ablation or inactivation of the amygdalae attenuate markedly analgesia afforded by systemic CB-1 agonists [42,43], which suggests that the amygdalae contribute significantly to cannabinoid analgesia.

We therefore hypothesised that the effect of THC on the affective qualities of pain in humans depends significantly on how the drug influences the functional activity of the amygdala. Since preclinical studies have shown that the cannabinoids are more potent in hyperalgesic states [28,40], we examined the effect of THC on ongoing pain and hyperalgesia that is induced by capsaicin sensitisation [34].

Capsaicin is an alkaloid derived from the chilli pepper. When applied topically, capsaicin produces ongoing burning pain as a result of peripheral sensitisation at the site of application [45]. In skin areas surrounding the site of capsaicin application, secondary punctate hyperalgesia occurs, which results from central sensitisation and is characterised by increased pain when normal tissue is mechanically stimulated [38,70]. Healthy cannabis-naive subjects volunteered for the randomised, double-blind, placebo-controlled, 2-way drug crossover study. The effects of THC (single dose) on the reported intensity and unpleasantness of capsaicin-induced pain and hyperalgesia were determined in individuals. Functional magnetic resonance imaging (fMRI) was used to investigate the extent to which the amygdalae explained the effects of THC on ongoing pain and hyperalgesia in individuals.

## 2 Methods

### 2.1 Subjects

Fifteen healthy, right-handed, cannabis-naïve men aged 24 to 34 years volunteered for the study. Women were excluded as the experimental drug study lasts about 4–6 weeks, and there is clear evidence for the potentially confounding effects of menstruation-related hormone fluctuations on pain in that month-long test period [59]. All subjects gave written informed consent and were familiarised with experimental procedures. The Mid and South Buckinghamshire local research ethics committee approved the study (LREC 06/Q1607/24).

Prior to entering the study, these subjects were screened using inclusion and exclusion criteria (Table 1). Data from 2 subjects were excluded despite negative recreational drug urinalysis because further interview during the course of the study revealed prior use of cannabis. One subject experienced drug-induced paranoia, which rendered him claustrophobic, and was excluded from the study because he could not undergo fMRI. His symptoms resolved completely over 24 hours and he remained well on follow-up at a year, with no further psychiatric symptoms.

### 2.2 Study design

The study was randomised, double blind, and placebo controlled. Each subject underwent 4 separate study sessions in a 2-by-2 factorial design. The factors were drug (15 mg THC or placebo taken orally; The Specials Laboratory, Northumberland, UK) and state (normal or capsaicin-induced sensitisation).

The sessions were balanced for order. To minimise wash-over effects related to THC or topical capsaicin application, the sessions were separated by at least a week and the active treatments for each study factor were administered on alternate sessions. Before each session, subjects fasted for 8 hours and abstained from caffeine and alcohol. Prior to drug ingestion (ie, baseline), subjects completed a simple visual-motor reaction time task (programmed using Delphi) [12] and provided a urine sample for drug screen (Multi-Drug Dip Card, Quantum Diagnostics, Waltham Abbey, UK). Subjects rested in a dedicated room for 2 hours post drug ingestion, after which the area for topical cream application and punctate mechanical stimulation was defined. A horizontal line was drawn over the skin on the anteromedial aspect of the right leg, 12–15 cm proximal to the medial malleolus. Two millilitres of 1% topical capsaicin (Propharma, Horsham, UK) or placebo cream was applied immediately above the line and occluded with clear film dressing (Tegaderm™, 3M Health Care, Neuss, Germany). The cream remained on the skin until the end of the fMRI session. The area, 4 × 4 cm, 1 cm below the horizontal line, was designated for punctate mechanical stimulation during fMRI.

Three hours post drug ingestion, fMRI was performed. The subjects repeated the simple reaction time task 3.5 hours post drug ingestion. Reaction time data from one subject was unavailable because of equipment failure. A 10-mL venous blood sample was then obtained for gas chromatography-mass spectrometry assay of plasma cannabinoid concentrations (ABS Laboratories, Hertfordshire, UK).

### 2.3 fMRI scanning and stimulation paradigm

Functional imaging data were acquired with a 3 Tesla human MRI system (1 m bore; Oxford Magnet Technology, Oxford, UK) using a transmit-receive birdcage radiofrequency coil and a high-performance reduced-bore gradient coil (Magnex SGRAD MK III, Oxford, UK) for signal detection. The T2^∗^-sensitive gradient echo-planar-imaging sequence with the following parameters was used: 3-second repetition time, 30-ms echo time, 41 contiguous 3-mm-thick slices, field of view 192 × 192 mm, matrix 64 × 64 pixels, 500 volumes. The first 4 volumes were discarded to permit equilibration of the blood-oxygen-level-dependent (BOLD) signal. Field maps were obtained using a symmetric-asymmetric spin-echo sequence (echo time 20 ms, 2.5 ms dwell time, field of view, and matrix identical to echo planar imaging). A T1-weighted structural image (1 mm^3^ voxel) was acquired to facilitate registration of T2^∗^-weighted images to standard stereotactic space (Montreal Neurological Institute [MNI] −152 template).

Once the subject was comfortably supine in the magnet bore, a single mean arterial pressure cuff measurement was obtained, and continuous monitoring of pulse rate, pulse oximetry (SpO_2_), end-tidal CO_2_ partial pressures (P_ET_CO_2_) (9500 Multigas Monitor, Wardray Premise, Surrey, UK) via nasal cannulae (Salter Labs, Arvin CA, USA), respiratory rate (RR), and chest excursions (Piezo Respiratory Belt Transducer, ADInstruments, Oxfordshire, UK) commenced. These on-line measurements helped ensure subject safety and provided cardiorespiratory data that were employed for several purposes. First, to correct for interregional correlations related to cardiorespiration (heart-rate and breathing fluctuations) during the analyses of functional connectivity. Second, to exclude any confound on baseline BOLD signal related to hypoxemia (decreased SpO_2_), or hypercarbia (increased CO_2_) related to excessive sedation caused by THC.

Just prior to the functional scans, the subject rated the intensity and unpleasantness of ongoing pain. The sequence of events during the functional scans were as follows: a 5-minute period, during which the subject performed no overt task but remained awake with eyes closed; a 30-second period that commenced when the subject was given a tactile cue (a tap on the leg) to rate the ongoing pain; a 15-minute period, during which the subject was instructed to direct attention, with eyes closed, to the punctate mechanical stimuli that were applied to the designated area on the right leg; a 1-minute period when the subject was again cued to rate the ongoing and provoked pain; finally, the subject viewed 5 30-second blocks of a black-and-white checkerboard flickering at 2 Hz that alternated with 5 30-second blocks of stationary fixation.

Punctate stimulation was applied using a handheld mechanical probe (flat tipped, 200 μm diameter) calibrated to deliver 512 mN force [52]. Twenty-one stimuli, each lasting 1 second, were delivered. The interstimulus interval was randomly jittered (10–40 seconds) to allow higher effective sampling of the BOLD signal following punctate stimulation.

Subjects rated the intensity and unpleasantness of ongoing and provoked pain using an electronic MR-compatible visual analogue scale (VAS) with visual feedback projected onto a screen visible through periscope goggles (left anchor: not at all; right anchor: intolerable). The word “intolerable” on the right anchor signified the ethical limit of pain that can be induced, beyond which the experiment must cease. The intensity of ongoing and provoked pain was defined as the degree of burning and sharp sensation, respectively. The unpleasantness of ongoing and provoked pain was defined in terms of how much the sensation bothered the subject. The subject was allowed 15 seconds to consider each scale and was specifically instructed to rate the overall experience, rather than focus on discrete provoked sensations.

### 2.4 Analyses of psychophysical and cardiorespiratory data

Statistical analyses were performed using SPSS 15.0 for Windows (SPSS Inc, Chicago, IL, USA). Repeated-measure 2 × 2 analysis of variance, with Greenhouse-Geisser correction for nonsphericity of data, was employed to ascertain significant effects of drug (THC or placebo) and state (normal or capsaicin sensitised) on the following parameters: intensity and unpleasantness of ongoing as well as provoked pain. Each of the 4 pain parameters was calculated as the mean of the 3 VAS ratings reported during fMRI scan. The repeated-measure analysis of variance was similarly employed to ascertain significant drug and state effects on simple reaction time. The effect of the drug on reaction time was measured as the difference between postdrug and baseline values. Bonferroni corrections were employed during post hoc tests. The effects of drug and state on the mean pulse rate, RR, SpO_2_, and P_ET_CO_2_ were similarly examined. A paired *t* test was used to compare plasma concentrations of THC and 11-hydroxy THC obtained from blood sampling 4 hours post dose.

### 2.5 Analyses of imaging data

fMRI analyses were performed using FEAT (FMRIB Expert Analysis Tool) Version 5.92, part of FSL (FMRIB’s Software Library, http://www.fmrib.ox.ac.uk/fsl).

Preprocessing included motion correction [30]; removal of nonbrain structures [58]; spatial smoothing using a Gaussian kernel of full width at half maximum 8 mm to reduce intersubject gyral differences; mean-based intensity normalisation of each 4-dimensional dataset by the same factor; high pass temporal filtering (Gaussian weighted least-squares straight line fitting, σ = 100 s); and physiological noise correction (cardiac and respiratory) using modified RETROICOR [7,22].

Once preprocessing was complete, the single time series of 496 volumes was divided into 3 time series. The first 126 volumes comprised the time series for the “no-task” period. The time series corresponding to punctate and visual stimulation comprised 215 and 106 volumes, respectively. Volumes corresponding to periods when subjects used the VAS were discarded.

Input stimulus functions, defined for the punctate and visual stimuli, were convolved with the canonical hemodynamic response function (mean lag 6 seconds, full width at half maximum 6 seconds) to yield regressors of interest for the general linear model. Individual time-series statistical analysis was carried out using FILM (FMRIB’s Improved Linear Model) with local autocorrelation correction [66]. Motion parameters acquired from preprocessing were included as nuisance regressors. First-level statistical maps were generated for BOLD activation associated with punctate and visual stimulation. The functional images were field-map corrected to reduce B_0_ distortion in the orbital-frontal and temporal regions [29]. Image registration to high-resolution structural and standard MNI-space images was then performed for the purposes of group analyses [30,31].

#### 2.5.1 Whole brain analyses

The aim of the whole brain analyses was to identify where THC affected brain activity that was associated with capsaicin-induced hyperalgesia. Group mean maps were generated using FLAME (FMRIB’s Local Analysis of Mixed Effects), a random-effects analysis [5,65]. A repeated-measures factorial analysis was used to generate *z* (Gaussianised T/F) statistic maps of the main (drug, state) and interaction (drug × state) effects on BOLD responses to punctate stimulation. The cluster-based correction for multiple voxel comparisons (*z* > 2.0, *P* < 0.05) was then applied to the whole brain map [17].

#### 2.5.2 Region of interest analyses

Region of interest (ROI) analyses were carried out to test our a priori hypothesis that amygdala activity contributes significantly to the analgesic effects of THC in humans, and to exclude global effects of THC on BOLD responses [64].

All anatomical ROIs were defined using the Harvard Oxford Cortical and Subcortical Structural Atlas (http://www.fmrib.ox.ac.uk/fsl/data/atlas-descriptions.html), which is a probabilistic population-based atlas. Only voxels estimated at >50% of probability of being in the structure were included in the ROI. The approach ensured a reproducible and conservative definition for the amygdala and intracalcarine (visual) anatomical ROI. The threshold of significance for small volume correction [67] for all ROIs was set to *P* < 0.01.

The cluster of voxels within the amygdala anatomical ROI on which THC had a significant main effect during hyperalgesia was chosen as the functional ROI. The percentage change in BOLD signal was extracted and averaged from significant voxels within the functional ROI using Featquery (http://www.fmrib.ox.ac.uk/fsl/feat5/featquery.html) for every subject and condition. Two-tailed significance testing of the Pearson’s *r* was then used to examine correlations between the drug-induced change in amygdala reactivity to noxious stimulation and the drug-induced change in the reduction of pain ratings.

#### 2.5.3 Interaction effects of THC and capsaicin on the functional connectivity of the amygdala

Functional connectivity (Fc) is a measure of correlated BOLD activity between the reference and target region(s). For this study, the reference ROI comprised the amygdala cluster of voxels on which THC had a significant main effect during hyperalgesia (ie, the functional ROI).

Converging lines of evidence suggest that Fc reflects anatomically and functionally relevant coupling within neuronal circuitries [50], with the caveat that a finding of “functional connection” does not prove structural or causal connections, as Fc measures are correlative in nature. Analyses of Fc in this study were achieved with methods developed to identify psychophysiological interactions [16]. The factorial experimental design employed in this study allowed isolation of Fc that relates specifically to the effects of THC on ongoing pain [23].

The first 126 volumes of the fMRI scan comprised the time series for the “no-task” period, during which the subjects performed no overt task except to stay awake with eyes closed whilst experiencing the effects of drug and capsaicin sensitisation. These time series were used for the Fc analyses. BOLD signals from all voxels comprising the amygdala functional ROI were extracted and averaged to produce the regressor for the general linear model of BOLD activity during the “no-task” period. Preprocessing of the fMRI time series was as described above. Motion parameters acquired from preprocessing were included as nuisance regressors. The first-level analyses of the general linear model served to generate whole-brain images of the functional connections of the right amygdala ROI for every subject and session. As before, the images were corrected for B_0_ distortion [29] before registration to high-resolution structural and standard MNI-space images was performed [30,31]. The transformed images were subsequently subjected to the group-level random-effects analysis [5,65]. A repeated-measures factorial analysis was used to generate *z* (Gaussianised T/F) statistical maps to represent the interaction effect (drug × state) on BOLD responses on the Fc of the amygdala ROI. For consistency, the cluster-based correction for multiple voxel comparisons (*z* > 2.0, *P* < 0.05) was again applied to the whole brain maps [17].

## 3 Results

**Fig. 1:**
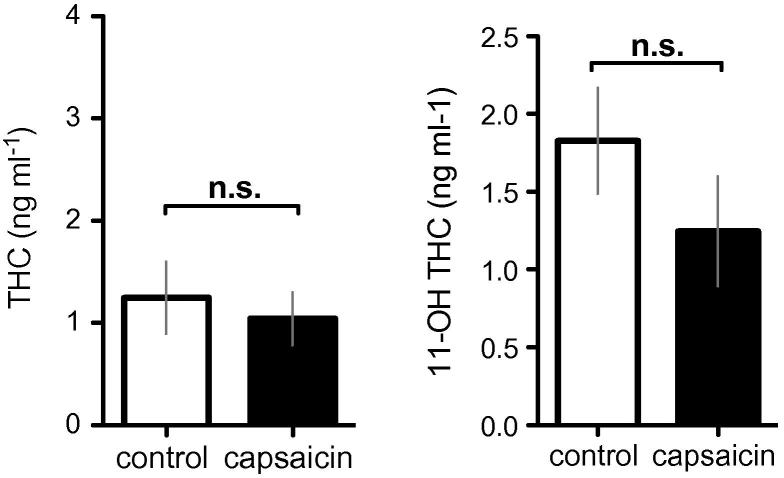
The plasma concentrations of delta-9-tetrahydrocannabinol (THC) and its active metabolite, 11 hydroxy (OH)-THC. Blood was sampled for plasma concentrations about 3.5 hours after THC was given. There were no significant differences (2-tailed paired *t* test, *P* > 0.05). Error bars represent SEM. Black and clear bars represent capsaicin and control sessions respectively. n.s., nonsignificant.

The data reported here are from 12 healthy, right-handed, cannabis-naïve men aged 24 to 34 years. All subjects underwent 4 experimental sessions in the crossover study, where THC or placebo (PLC) was administered on normal or capsaicin-sensitised skin. The plasma concentrations of THC and 11-hydroxy THC obtained from blood sampling 4 hours post dose were not significantly different (paired *t* test; *P* > 0.1) between control and capsaicin conditions (Fig. 1).

### 3.1 Behavioral and physiological effects of THC

**Fig. 2:**
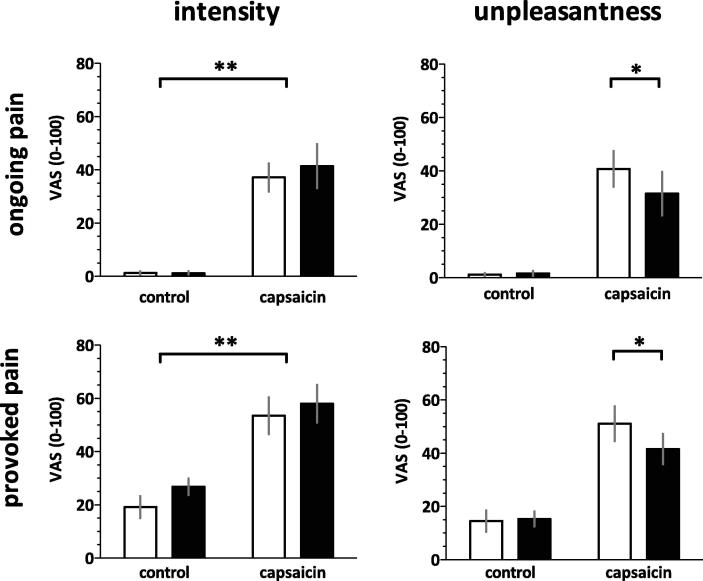
Capsaicin induced significant ongoing burning pain and caused an increase in pain provoked by punctate stimuli. Compared to placebo, delta-9-tetrahydrocannabinol (THC) significantly reduced the effect of capsaicin on the unpleasantness, but not the intensity of provoked and ongoing pain (Bonferroni adjusted post hoc paired *t* test, ^∗^*P* < 0.05). Error bars represent SEM. Black and clear bars represent THC and placebo, respectively. VAS, visual analogue scale.

Capsaicin induced ongoing burning pain [main effect: intensity, *P* < 0.001, F(1, 11) = 38.0] and heightened pain provoked by noxious punctate stimuli [main effect: intensity, *P* < 0.001, F(1, 11) = 26.5] (Fig. 2, Table 2). Compared to PLC, THC did not significantly alter the perceived intensity of ongoing or mechanically provoked pain. However, the cannabinoid significantly reduced the perceived unpleasantness of both ongoing and provoked pain after capsaicin sensitisation (2-tailed paired *t* tests, Bonferroni adjustment: *P* < 0.05).

**Fig. 3:**
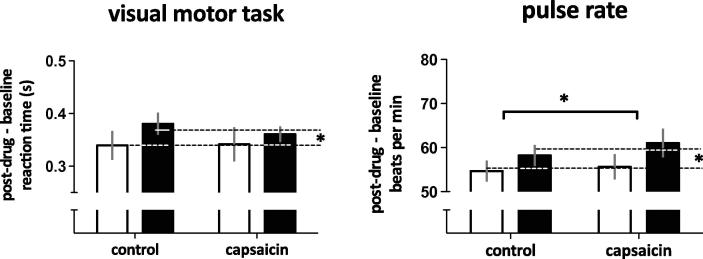
Delta-9-tetrahydrocannabinol (THC) slowed reaction times (main effect, ^∗^*P* < 0.05) and increased resting pulse rate slightly (main effect, ^∗^*P* < 0.05). There was no significant effect of capsaicin on reaction times. However, there was a slight increase in resting pulse rate during capsaicin-induced sensitisation compared to control (main effect, ^∗^*P* < 0.05). Error bars represent SEM. Black and clear bars represent THC and placebo, respectively.

Compared to PLC, THC slowed the response to a simple visual-motor reaction-time task [main effect: *P* < 0.05, F(1, 10) = 5.9], which was consistent with its sedative effects. Capsaicin did not affect reaction times (Fig. 3, Table 2).

### 3.2 Effects of THC on brain activity related to capsaicin-induced hyperalgesia

**Fig. 4:**
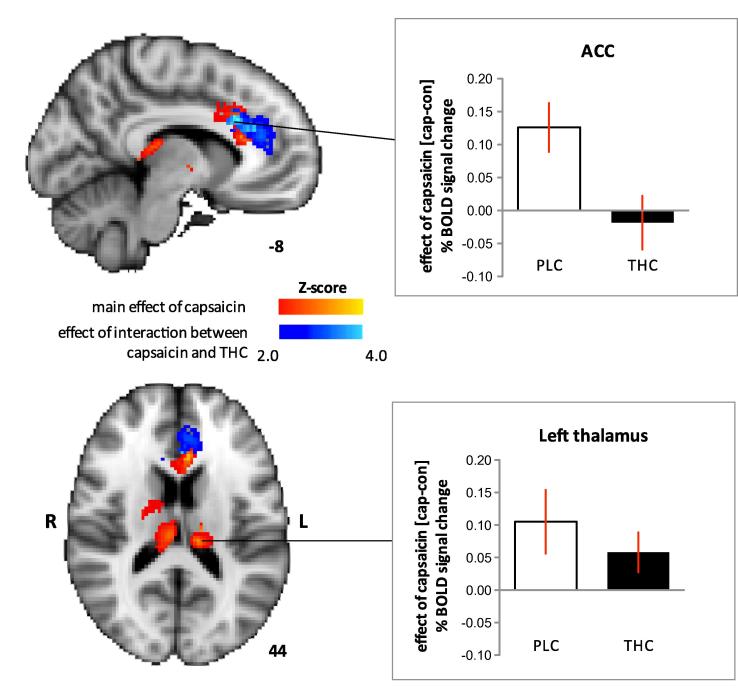
The main effect of capsaicin, indicated in red, was to increase blood-oxygen-level-dependent (BOLD) activation in the anterior cingulate cortex (ACC) (Montreal Neurological Institute [MNI] peak coordinates −6, 20, 30; *z* score = 3.8) and thalami (left thalamus: MNI peak coordinates −12, −26, 16; *z* score = 3.2, right thalamus: MNI peak coordinates 10, −22, 14; *z* score = 3.6). The effect of interaction between delta-9-tetrahydrocannabinol (THC) and capsaicin, indicated in blue, was significant in the ACC only (MNI peak coordinates −8, 22, 28; *z* score = 4.6). The graphs clarify the effects of THC and placebo (PLC) on capsaicin-induced BOLD responses. Capsaicin-induced BOLD response was calculated as the difference (cap − con) in percentage BOLD signal change between capsaicin (cap) and control (con) sessions. Compared to PLC, THC decreased the BOLD response in the ACC related to hyperalgesia (top graph). In contrast, activation within thalami related to hyperalgesia did not differ significantly. Coloured bars denote range of *z* scores. Clear and black bars represented PLC and THC, respectively. Error bars represent SEM.

Capsaicin sensitisation increased BOLD activity during provoked pain in the thalami, as well as the anterior cingulate cortex (ACC; main effect of capsaicin, *z* > 2.0; cluster-based correction *P* < 0.05) (Fig. 4). Please refer to Table 3 for the local maxima of activations in other brain regions for the effects related to capsaicin-induced punctate hyperalgesia that did not survive cluster-based threshold (*P* < 0.05) for multiple comparisons. However, a significant interaction between capsaicin and THC on BOLD activation occurred in the ACC only (*z* > 2.0; cluster-based correction *P* < 0.05). Activation in the ACC related to capsaicin-induced hyperalgesia was decreased with THC compared to PLC. The extent to which this finding corroborates the effect of THC on the unpleasantness of capsaicin-induced hyperalgesia is discussed later.

**Fig. 5:**
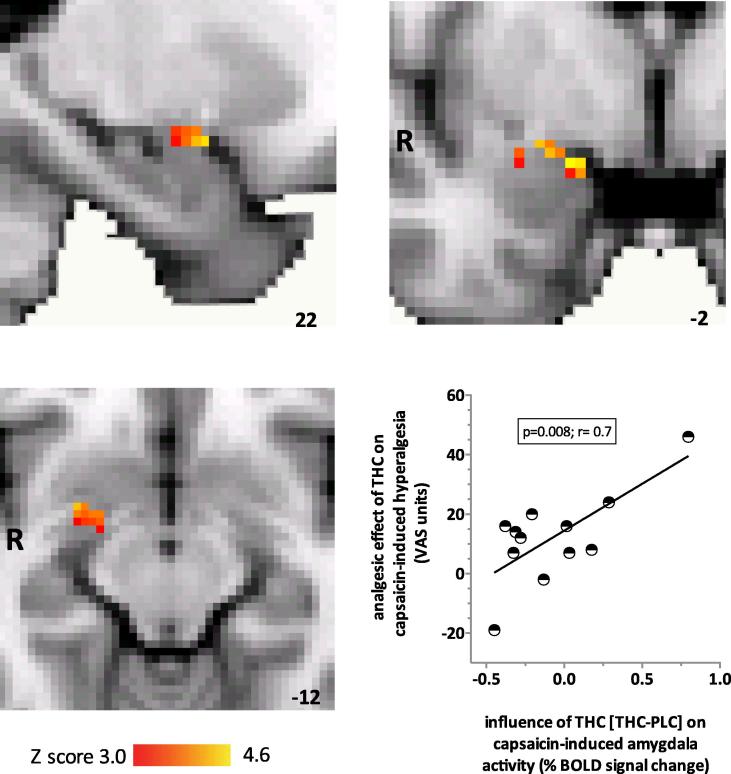
Region-of-interest analyses revealed that the main effect of delta-9-tetrahydrocannabinol (THC) was to significantly increase blood-oxygen-level-dependent (BOLD) response within the right amygdala (37 significantly activated voxels; Montreal Neurological Institute [MNI] coordinates of peak voxel 26, 0, −14; *z* score = 3.23). The coloured bars represent the range of *z* scores. MNI coordinates are indicated at the bottom right of each slice. The graph shows that in individuals, analgesic effect of THC on the unpleasantness of hyperalgesia was positively and significantly correlated with the effect of THC compared to placebo (PLC) on capsaicin-induced responses in the right amygdala (*P* < 0.01, *r* = 0.72). VAS, visual analogue scale.

Next, we focused on the amygdalae as ROIs, given our hypothesis regarding their role in cannabinoid analgesia [42,43]. We found that the main effect of THC was a significant increase in right amygdala BOLD response to noxious stimulation (small volume correction, *P* < 0.01) (Fig. 5). In order to ascertain whether the influence of THC on right amygdala BOLD response was relevant to the effect of THC on the unpleasantness of capsaicin-induced hyperalgesia, we examined the correlation between the BOLD response and the effect of the drug on the unpleasantness of hyperalgesia in individuals. We found a significantly positive correlation (*P* < 0.01, *r* = 0.72) between the effect of THC on the right amygdala BOLD response and the analgesic effect of THC (Fig. 5), which was defined as the reduction in the unpleasantness of capsaicin-induced hyperalgesia.

In contrast, there were no significant effects of THC or capsaicin on BOLD responses related to visual stimulation in the intracalcarine anatomical-region ROI. The lack of drug effect on the visual-related BOLD responses activity concurs with results from a previous fMRI study [46] and suggests a specific effect of THC on BOLD responses that are related to the neural processing of noxious inputs.

We further confirmed that there was no significant influence of either THC or capsaicin on P_ET_CO_2_, RR, or SpO_2_ because these parameters can influence BOLD activity directly. THC increased pulse rate [main effect: *P* < 0.05, F(1, 11) = 5.0], as did topical capsaicin [main effect: *P* < 0.05, F(1, 11) = 6.2] (Fig. 3, Table 2). However, the drug effects on pulse rate were slight (3–5 beats per minute) and did not exhibit any interaction effects with capsaicin that could account for our observed effects of THC on the BOLD response during capsaicin-induced sensitisation.

### 3.3 Effects of THC and capsaicin on the Fc of the right amygdala

**Fig. 6:**
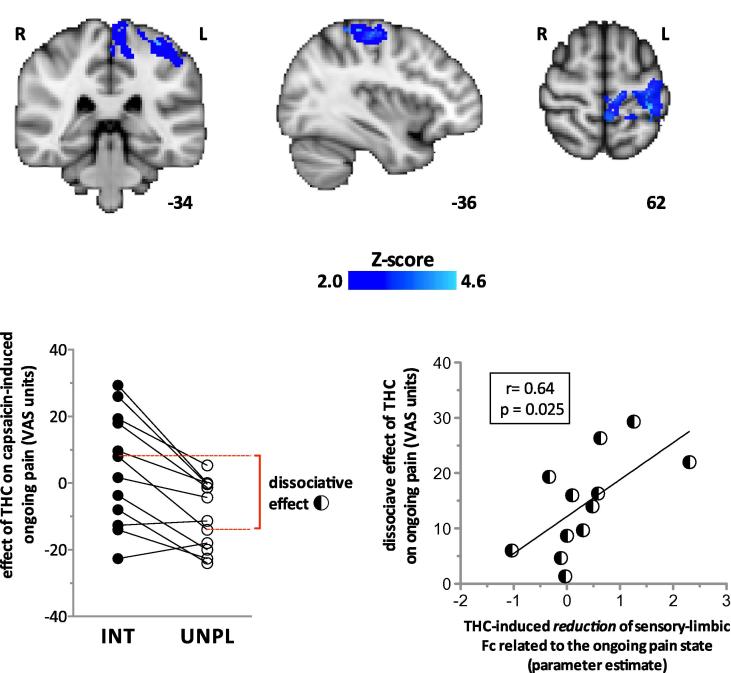
(Top) Delta-9-tetrahydrocannabinol (THC) significantly reduced the right amygdala and primary sensorimotor functional connectivity (Fc) during capsaicin-induced ongoing pain (*z* > 2.0; cluster-based correction). *z*-Scores indicating the degree to which THC reduced Fc are scaled in blue. Montreal Neurological Institute (MNI) coordinates are indicated at the bottom right of each slice. (Bottom-left graph) The dissociate effect that THC has on the intensity (INT) and unpleasantness (UNPL) of capsaicin-induced pain in each individual (represented by connecting lines). Negative visual analogue scale (VAS) units on the Y-axis indicate an analgesic effect. The dissociative effect of THC on pain was calculated as the absolute difference of drug effects on the intensity and unpleasantness of ongoing pain, and is indicated in dashed red lines and bracket for an individual. (Bottom-right graph) THC-induced reduction of sensory-limbic Fc was positively correlated with the dissociative effect of the drug on ongoing pain. The interaction term for the drug effect on the sensory-limbic Fc associated with ongoing pain is drug [PLC-THC] × state [CAP-CON]. For each individual, parameter estimates for the interaction effect were calculated for the left-S1 region of interest, which consisted of an 8-mm-diameter sphere centred on the group peak interaction (state × drug) effect within the post-central gyrus (MNI coordinates −34, −36, 62). PLC, placebo; CAP, capsaicin-sensitised state; CON, control state.

Having isolated the region in the right amygdala involved in the effect of THC on capsaicin-induced hyperalgesia, we proceeded to identify the functional connections underlying the analgesic effect on capsaicin-induced ongoing pain. We found that THC significantly reduced (*z* > 2.0; cluster-based correction *P* < 0.05) Fc between the right amygdala and the primary sensorimotor cortex during the ongoing pain state (Fig. 6, top images). Because THC significantly suppressed BOLD responses within the ACC during punctate hyperalgesia (Fig. 4), we also examined whether Fc between the right amygdala and that specific ACC region was altered during ongoing pain. The results suggest that THC increased Fc between the right amygdala and ACC, but did not survive voxel-based (small-volume) correction (*P* < 0.01) [67].

We then explored to what extent the decreased Fc between the amygdala and sensorimotor cortex induced by THC was related to the psychophysical effects of the drug on ongoing pain. In the present study, THC had divergent effects on the intensity and unpleasantness of ongoing pain. Individuals who experience a reduction in the unpleasantness of ongoing pain may experience either similar or opposite effects on the intensity of pain (Fig. 6, bottom-left graph). This dissociative effect of THC on pain was quantified by the absolute difference between the effects of the drug on the intensity and unpleasantness of ongoing pain (Fig. 6, bottom left).

We investigated whether the dissociative effects of THC on the experience of pain might be explained by altered Fc between the amygdala and primary somatosensory cortex (S1) specifically. The sensation of pain was induced by application of capsaicin in the right lower limb. Hence, the S1 ROI comprised an 8-mm sphere centred on the voxel with the group peak interaction (state × drug) effect (MNI coordinates −36, −34, 62; *z* = 4.1) within the left postcentral gyrus (as defined by the Harvard Oxford Cortical Structural Atlas (http://www.fmrib.ox.ac.uk/fsl/data/atlas-descriptions.html). Consistent with other ROIs defined using this approach in the study, only voxels estimated at >50% of probability of being in the structure were included in the ROI.

The parameter estimates for the interaction effect (drug × state) that represents the reduction of Fc between the right amygdala and primary somatosensory area (S1) of THC during ongoing pain were then derived for each individual (http://www.fmrib.ox.ac.uk/fsl/feat5/featquery.html). We found that the reduction of sensory-limbic Fc by THC was positively correlated (Pearson’s *r* = 0.64, *P* < 0.05) with the divergent effects of the drug on the intensity and unpleasantness of ongoing pain (Fig. 6, bottom-right graph). In other words, the greater the effects of THC on one aspect of pain relative to the other, the greater the loss of Fc between the amygdala and S1 (primary somatosensory area).

## 4 Discussion

Previous psychophysical studies have revealed inconsistent results regarding the effects of THC on pain and hyperalgesia [1,35,56,62]. Such studies employed psychophysical indices that focused on the sensory aspects of pain, and consistent with these studies, we report no significant effect of THC on the perceived pain intensity (sensory aspect) of capsaicin-induced pain or hyperalgesia. Instead, we found that THC relieved specifically the perceived unpleasantness of pain and hyperalgesia induced by capsaicin. The divergent effect of THC on sensory and affective aspects of somatosensation has also been reported by Libman and Stern, who observed that THC can lower sensory thresholds to innocuous stimuli whilst increasing pain tolerance [41]. THC does have sedative effects [12], which was evidenced by a generalised increase in visual-motor reaction times in our study. Importantly, the analgesic effect of THC was evident only when pain and hyperalgesia were induced by capsaicin. Thus, general sedation does not account for the analgesic effect of THC that was reported by our subjects.

The dissociative analgesic effects of THC on experimentally induced pain in this study were further corroborated by fMRI data. We found that THC specifically decreased BOLD activation related to hyperalgesia within the ACC but did not significantly reduce activation in the posterior thalami (Fig. 4). Only a subset of all regions known to be involved in punctate hyperalgesia was significantly activated in this study when compared to 2 of our previous studies [38,69]. This may relate to the presence of ongoing pain induced by highly concentrated capsaicin (1%) that was maintained throughout scanning and was not present in those previous studies. Ongoing pain induced by capsaicin itself is associated with increased cerebral blood flow and hence, elevates baseline BOLD signal in brain regions associated with punctate hyperalgesia [27]. Experimental data have revealed that the absolute increase in BOLD signal evoked by a stimulus is constant regardless of the baseline signal [26,57]. Increases in the baseline BOLD signal can be associated with a relative decrease of stimulus-evoked response compared to the baseline. This implies that areas where the increase in activity related to punctate hyperalgesia is less marked to begin with may not exhibit significant responses when the baseline signal is increased in the presence of ongoing pain. Hence, BOLD-fMRI assay sensitivity in our study may be reduced for the detection of responses related to hyperalgesia with strong ongoing pain. The robustness of BOLD responses in the anterior cingulate and thalami to punctate stimulation in the presence of ongoing pain suggests their significant involvement during capsaicin-induced hyperalgesia, which is supported by previous data of capsaicin-induced hyperalgesia (refer to Supplementary Table in [38]).

Preclinical studies consistently demonstrate that ACC lesions reduce conditioned place avoidance but not withdrawal behaviour in inflammatory and neuropathic [10,19,33,37] pain models, supporting a role for that region in higher-order pain behaviour. Patients who have undergone anterior cingulotomy for the treatment of intractable pain [25,63,68] have relatively intact sensory perception of noxious stimuli, but their affective responses to the stimulus are markedly attenuated [15]. In further support of the role of the ACC for the affective-motivational aspects of pain, Rainville and colleagues have shown that the limbic structure is more active when the unpleasantness of pain is aggravated by hypnotic suggestion [49]. Notably, the ACC region activated in their study is precisely where THC suppressed activation related to hyperalgesia in our study.

Based on recent cytoarchitectural studies, Vogt suggests that the ACC may be subdivided into the anterior and mid cingulate cortices [60]. Under his proposed anatomical classification, the cingulate region in our study is identified as the anterior mid cingulate cortex, which crucially, is a region that communicates anatomically with the amygdala [3,20,61]. Preclinical studies clearly demonstrate that ablation or inactivation of the amygdalae attenuate analgesia afforded by systemic CB-1 agonists [42,43]. These data led us to specifically explore whether amygdala activity plays a key role during cannabinoid analgesia in humans. In contrast to the decreased ACC response during cannabinoid analgesia, the main effect of THC was to increase amygdala response during noxious stimulation. Importantly, the increase in capsaicin-induced amygdala response and the analgesic effect of THC was positively correlated, which indicates that amygdala activity contributes to interindividual variation in analgesic drug response. Evidence from rodent studies suggests that cannabinoid analgesia involves activation of the brainstem circuitry for the descending inhibition of nociception via the amygdala [42,44]. However, our Fc analyses did not reveal significantly altered Fc between the amygdala and brainstem during cannabinoid analgesia with this study design.

Instead, we found that THC significantly reduced Fc between the amygdala and a large portion of the primary sensorimotor area. Specifically, the reduction of Fc between the amygdala and the left S1 region was positively correlated with the dissociative effects of the drug on the reported intensity and unpleasantness of pain in the right lower leg. For an unbiased analysis, the S1 ROI was centred on the peak coordinates within the left postcentral gyrus, even though they are unlikely to fall within the representation area of the right leg. Direct anatomical connections between S1 and amygdalae are sparse [55]. In contrast, ample clinical [15,48] and physiological [18,24,36,49] evidence in humans supports the concept that nociceptive information ascends in divergent parallel ascending pathways to sensory and limbic regions. A selective action of THC on CB-1 receptor dense limbic pathway could explain parsimoniously the findings of reduced sensory-limbic Fc in the face of drug-induced dissociative analgesia. However, the parallel model does not account for how the separable dimensions of pain are normally united in human consciousness [14], and THC might well disrupt the integration of neural information between sensory and limbic regions from which the experience we recognise as pain arises.

We found involvement of the right rather than the left amygdala during cannabinoid analgesia. There are previous data that can account for the lateralisation of cannabinoid effects on pain-related amygdala activity. Several investigators have recently demonstrated that inflammation-induced activation or plasticity of the right amygdala is independent of the laterality of peripheral inflammation, which attests to the right-hemispheric lateralisation of amygdala function during pain [11,32]. The male gender in our study may also contribute to the hemispheric lateralisation of drug effect: highly salient stimuli are preferentially processed by the right amygdala in men [9].

THC was administered systemically in our study. Consequently, peripheral mechanisms for the observed effect of the cannabinoid on capsaicin-induced hyperalgesia cannot be excluded [53]. However, peripheral or spinal mechanisms do not account adequately for the divergent effects of THC on the sensory and affective aspects of pain that we observed in this study. Instead, our data demonstrate that the effects of THC in the brain, characterised by altered functional activities of the right amygdala, account for the interindividual variation in analgesic efficacy.

Cannabis has been reported to be remarkably effective for the relief of otherwise intractable pain in patients [54]. However, the bases of such pain relief afforded by this psychotropic agent are debatable [51,54]. Our data reveal that specific effects of THC, the psychotropic component of cannabis, on the amygdala contribute to the analgesic effects of the drug reported by healthy volunteers. Identifying patients who rely on similar central effects from cannabis-based medicines for pain relief remains challenging, but our study indicates that the use of functional brain imaging for that purpose merits further investigation.

## Conflict of interest statement

No conflicts of interest are declared.

## Figures and Tables

**Table 1:** Study inclusion, exclusion, and discontinuation criteria.

Inclusion criteria
1. Male subjects
2. Age between 18 and 50 years
3. Body weight between 60 and 100 kg and within the normal range for height (assessed by Quetelet index), normal range 18–24 kg m^−2^
4. Cannabis naïve
5. Nonsmokers
Exclusion criteria
1. Evidence of any clinically significant disease on direct questioning and physical examination with particular attention to cardiovascular disorder, chronic pain, psychiatric or neurological condition
2. History of clinically significant drug allergies (including allergy to chilli peppers)
3. Report of taking, or having taken, any prescribed or over-the-counter drug in the week prior to the start of the study
4. Received any experimental drug in the last 3 months prior to the first dosing day of the study
5. Habit of drinking more than 28 units of alcohol per week
6. Evidence of past or current recreational drug use on direct questioning; or positive drug urinalysis on prestudy screening
7. Contraindication to fMRI
Discontinuation criteria
1. Ineligibility, either arising during the study or retrospectively having been overlooked during screening
2. Subject noncompliance with study requirements (for example, a positive urinalysis prior to any of the study sessions)
3. Any adverse event, which results in the subject being unable to continue to comply with study procedures

fMRI, functional magnetic resonance imaging.

**Table 2:** Repeated-measures 2 × 2 ANOVA (Greenhouse-Geisser correction) for the effects of drug (placebo or THC) and state (normal or capsaicin sensitised) on pain ratings, reaction time, and cardiorespiratory variables.

	*df* (hypothesis, error)	MS	F	*P*-value
Ongoing pain				
Intensity				
Drug (main effect)	1, 11	0.52	0.61	0.452
State (main effect)	1, 11	173.97	37.91	< 0.001
Drug x State (interaction)	1, 11	0.56	0.935	0.354
Unpleasantness				
Drug (main effect)	1, 11	2.32	7.567	0.019
State (main effect)	1, 11	144.94	24.88	< 0.001
Drug x State (interaction)	1, 11	2.8	11.11	0.007

Provoked pain				
Intensity				
Drug (main effect)	1, 11	4.56	3.34	0.095
State (main effect)	1, 11	128.5	26.5	< 0.001
Drug x State (interaction)	1, 11	0.3	0.3	0.595
Unpleasantness				
Drug (main effect)	1, 11	1.3	0.931	0.355
State (main effect)	1, 11	104.73	24.05	< 0.001
Drug x State (interaction)	1, 11	6.68	5.59	0.03
Visual motor reaction time				
Drug (main effect)	1, 10^a^	0.01095	5.863	0.036
State (main effect)	1, 10^a^	0.001131	1.312	0.2787
Drug x State (interaction)	1, 10^a^	0.001677	2.219	0.1672
Pulse rate				
Drug (main effect)	1, 11	172.1	4.912	0.0487
State (main effect)	1, 11	81.39	6.246	0.0296
Drug x State (interaction)	1, 11	0.0003	<<<1	0.9973
Respiratory rate				
Drug (main effect)	1, 11	1.647	0.7644	0.4006
State (main effect)	1, 11	1.365	0.1830	0.6711
Drug x State (interaction)	1, 11	3.321	0.6351	0.4424
End-tidal carbon dioxide				
Drug (main effect)	1, 11	18.40	3.454	0.09
State (main effect)	1, 11	9.541	1.711	0.2175
Drug x State (interaction)	1, 11	2.347	0.4247	0.5284
Pulse oxygen saturation				
Drug (main effect)	1, 11	2.093	0.9463	0.336
State (main effect)	1, 11	0.2765	0.1250	0.7253
Drug x State (interaction)	1, 11	2.093	0.9463	0.3360

ANOVA, analysis of variance; THC, delta-9-tetrahydrocannabinol; MS, mean square; *df*, degrees of freedom.

a Data unavailable for 1 subject because of equipment failure.

**Table 3:** Main effect of THC (*z* score >2.0) on BOLD activations in regions known to be involved in hyperalgesia that did not survive cluster-based correction for multiple comparisons (*P* < 0.05).

Region defined by the Harvard-Oxford structural probabilistic atlas (*P* > 0.5) L: left; R: right	MNI coordinate of peak voxel			Voxel level
	x	y	z	*z*-score
Brainstem (periaqueductal gray)	−6	−38	−24	3.4
Cerebellum	−16	−38	−24	4.12
L insula	−36	16	−12	3.65
R insula	38	−4	4	2.12
L primary somatosensory cortex	−50	−20	42	2.51
L amygdala	−26	10	14	2.58
R amygdala	26	0	−14	3.23
L hippocampus	−18	−14	−16	2.85
R hippocampus	24	−28	−8	2.89
L putamen	−26	0	−8	2.3
R putamen	24	4	−8	4.7
L caudate	16	−6	20	2.12

THC, delta-9-tetrahydrocannabinol; BOLD, blood-oxygen-level-dependent; MNI = Montreal Neurological Institute. The regions were defined using the Harvard-Oxford structural probabilistic atlas. The MNI coordinates of local maxima for each region are reported.
